# Patient Information Websites About Medically Induced Second-Trimester Abortions: A Descriptive Study of Quality, Suitability, and Issues

**DOI:** 10.2196/jmir.6380

**Published:** 2017-01-10

**Authors:** Tommy Carlsson, Ove Axelsson

**Affiliations:** ^1^ Department of Women’s and Children’s Health Uppsala University Uppsala Sweden; ^2^ Centre for Clinical Research Sörmland Uppsala University Eskilstuna Sweden

**Keywords:** consumer health information, induced abortion, information literacy, Internet, popular works, second pregnancy trimester

## Abstract

**Background:**

Patients undergoing medically induced second-trimester abortions feel insufficiently informed and use the Web for supplemental information. However, it is still unclear how people who have experience with pregnancy termination appraise the quality of patient information websites about medically induced second-trimester abortions, whether they consider the websites suitable for patients, and what issues they experience with the websites.

**Objective:**

Our objective was to investigate the quality of, suitability of, and issues with patient information websites about medically induced second-trimester abortions and potential differences between websites affiliated with the health care system and private organizations.

**Methods:**

We set out to answer the objective by using 4 laypeople who had experience with pregnancy termination as quality assessors. The first 50 hits of 26 systematic searches were screened (N=1300 hits) using search terms reported by the assessors. Of these hits, 48% (628/1300) were irrelevant and 51% (667/1300) led to websites about medically induced second-trimester abortions. After correcting for duplicate hits, 42 patient information websites were included, 18 of which were affiliated with the health care system and 24 with private organizations. The 4 assessors systematically assessed the websites with the DISCERN instrument (total score range 16-80), the Ensuring Quality Information for Patients (EQIP) tool (total score range 0-100), as well as questions concerning website suitability and perceived issues.

**Results:**

The interrater reliability was 0.8 for DISCERN and EQIP, indicating substantial agreement between the assessors. The total mean score was 36 for DISCERN and 40 for EQIP, indicating poor overall quality. Websites from the health care system had greater total EQIP (45 vs 37, *P*>.05) and reliability scores (22 vs 20, *P*>.05). Only 1 website was recommended by all assessors and 57% (24/42) were rated as very unsuitable by at least one assessor. The most reported issues with the websites involved lack of information (76%, 32/42), and poor design (36%, 15/42).

**Conclusions:**

The high number of irrelevant hits and poor quality of patient information websites are considerable issues that must be addressed and considered when consulting patients awaiting medically induced second-trimester abortions. In clinical encounters, health professionals should initiate discussions concerning websites about medically induced second-trimester abortions and inform patients about the issues and quality deficits associated with these websites.

## Introduction

Patients often use the Web to read about health-related information [1,2], particularly young [1,3] and pregnant women [4]. The Web is a potential source of highly accessible and tailored information that transcends traditional informational processes. Moreover, it has the potential to promote equity and patient empowerment by improving knowledge [5-8]. Many view the Web as an important source of health-related information [1], and patients use it before and after consultations with health professionals. Before a consultation, they use it to manage their situation and to decide for themselves whether or not they need professional help. After a consultation, they use it to feel reassured by reading supplemental information or due to dissatisfaction with the information offered during the clinical encounter with health professionals [9]. However, the considerable plethora of available and uncontrolled information on the Web results in disorganization, which can lead to difficulties navigating and finding relevant information [5,10]. Studies investigating techniques for information retrieval among lay consumers have observed suboptimal search methods when they try to find health-related information on the Web, suggesting a need for efforts to help patients identify high-quality information [11,12]. Moreover, the lack of peer reviews or other regulating systematic activities as methods to control the quality of the information available on the Web increases the risk of patients to come in contact with low-quality information [5], and the lack of easy access to high-quality, relevant information is an identified barrier for patients [10]. Consequently, poor quality of patient information websites is a concern [5,13,14]. Considering these problems and risks, research is needed to investigate the quality of patient information websites.

Individuals who undergo medically induced second-trimester abortions experience both emotional distress and physical pain before, during, and after the abortion [15-19]. To deal with their difficult situation, they have a great need for information and support [15-18]. Preparatory information is an indicator of quality abortion care, and the literature suggests that women should always be offered sufficient information before the procedure [20]. However, people who undergo the procedure describe that they feel insufficiently informed about it, resulting in unanswered questions and unpreparedness [15-18]. To feel adequately prepared and well-informed, they search the Web for supplemental information about pregnancy terminations [17,18,21]. However, inductive qualitative research indicates issues with searching difficulties and poor website quality [18], calling attention to the need for systematic investigations that draw more generalizable conclusions. Previous investigations on websites about abortions have shown that some of these websites include medical inaccuracies and misleading information [22-24], for example, erroneous claims about associations between abortion and mental health risks, preterm births, breast cancer, and infertility [22,24]. However, the quality of patient information websites about medically induced second-trimester abortions remains unclear.

Studies that investigate the quality of patient information on the Web typically use health professionals or researchers as assessors. However, previous research has illustrated a mismatch between patients’ perspectives and those of researchers [25] as well as health professionals [26], thereby raising questions concerning patient perspectives and preferences regarding patient information on the Web. In other words, it is possible that the perspectives and preferences regarding information websites differ among those consuming the information and those typically developing or investigating it. To accomplish high-quality patient information materials, research suggests a need for information developers to produce materials based on the needs of the intended consumers and to actively involve them in the development process [27]. It is still unclear how people who have experience with pregnancy termination appraise the quality of information websites about medically induced second-trimester abortions, whether they consider the websites suitable for patients, and what issues they experience when reading the information. Moreover, the mismatched perspectives call attention to the importance of surveying the landscape on the Web with regard to website affiliation in order to gain knowledge about the potential differences in quality between websites affiliated with the health care system and private organizations.

With this study, we set out to provide descriptive data on currently available patient information websites about medically induced second-trimester abortions, grounded in the perspectives and preferences of laypeople with personal experience. Thus, the aim was to investigate the quality of, suitability of, and issues with patient information websites about medically induced second-trimester abortions, and investigate possible differences between websites affiliated with the health care system and private organizations.

## Methods

### Study Context

In Sweden, all pregnant women are offered a second-trimester routine obstetric ultrasound examination, usually performed at 18 weeks of gestation. Swedish law states that pregnant women have the right to decide on termination of pregnancy up to 18 completed weeks of gestation. At later gestations, approval must be granted for from the National Board of Health and Welfare, and in practice, few pregnancies are terminated after 22 weeks. The majority of second-trimester abortions performed in Sweden are medically induced labors with vaginal deliveries of the fetus [28].

### Search Procedure

Thirteen search terms reported by 4 individuals who have experience with medically induced second-trimester abortion were used to find Swedish websites about medically induced second-trimester abortions. Bing and Google, currently the 2 most used search engines on the Web [29], were used to perform the searches. The searches were conducted in October 2015 and yielded a total of 4,578,500 hits (Table 1).

**Table 1:** Search terms and number of hits.

Search terms	Bing	Google
Abortion due to a heart defect	16,900	11,500
Angel mum	36,600	44,100
Grieving work after losing a baby	45,800	56,800
How a late abortion is done	1,770,000	212,000
How great is the risk that a fetal heart defect is repeated	16,900	6490
How to manage a late abortion	19,400	123,000
Late abortion	81,700	454,000
Late abortion after discovery in routine ultrasound	63,300	132,000
Late termination of pregnancy	12,800	8610
Miscarriage after late abortion	13,100	119,000
Pregnant after late abortion	174,000	193,000
Termination	194,000	618,000
Termination in week 20	72,100	83,400
Total hits (all search terms)	2,516,600	2,061,900

**Figure 1:**
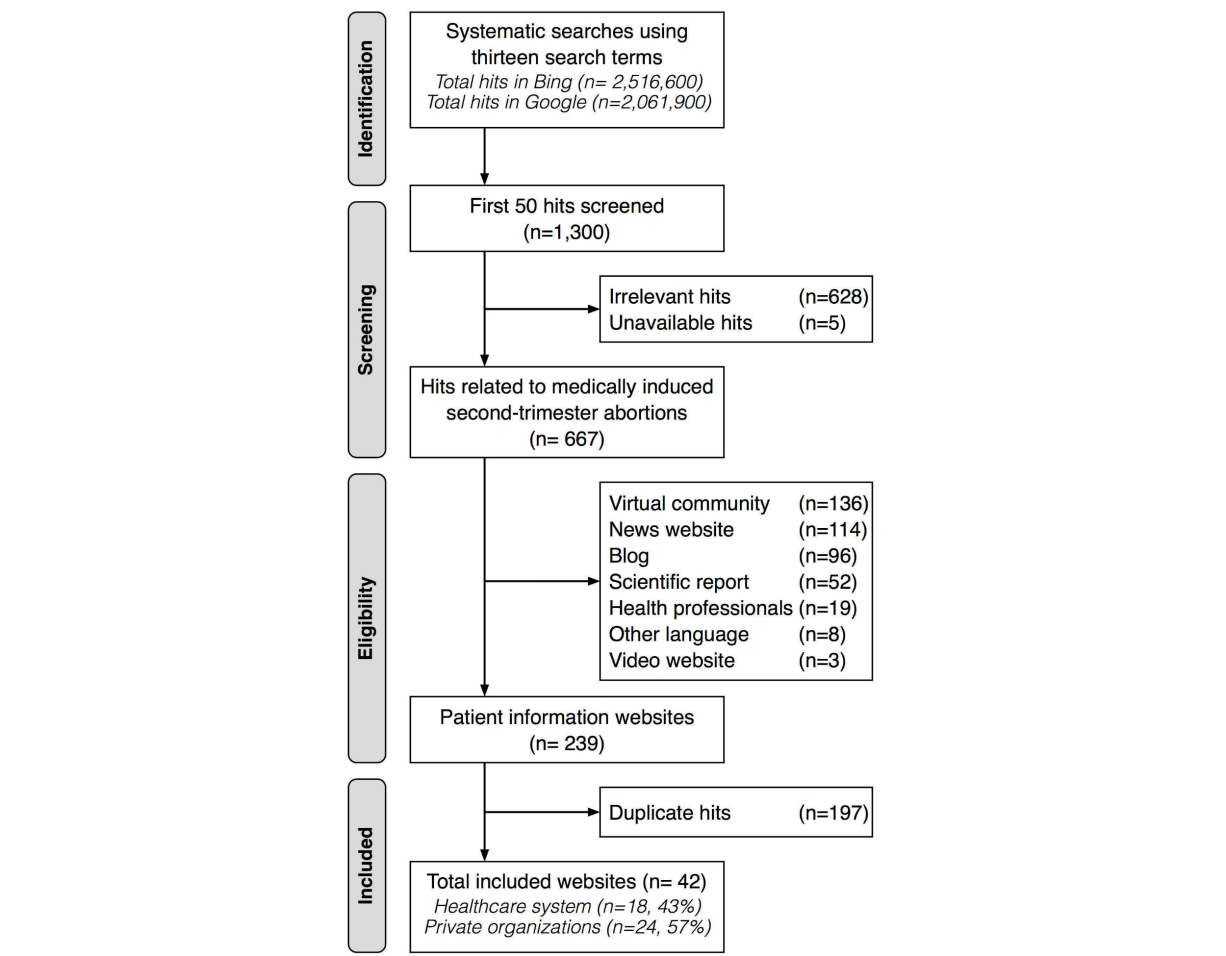
Sampling procedure.

**Figure 2:**
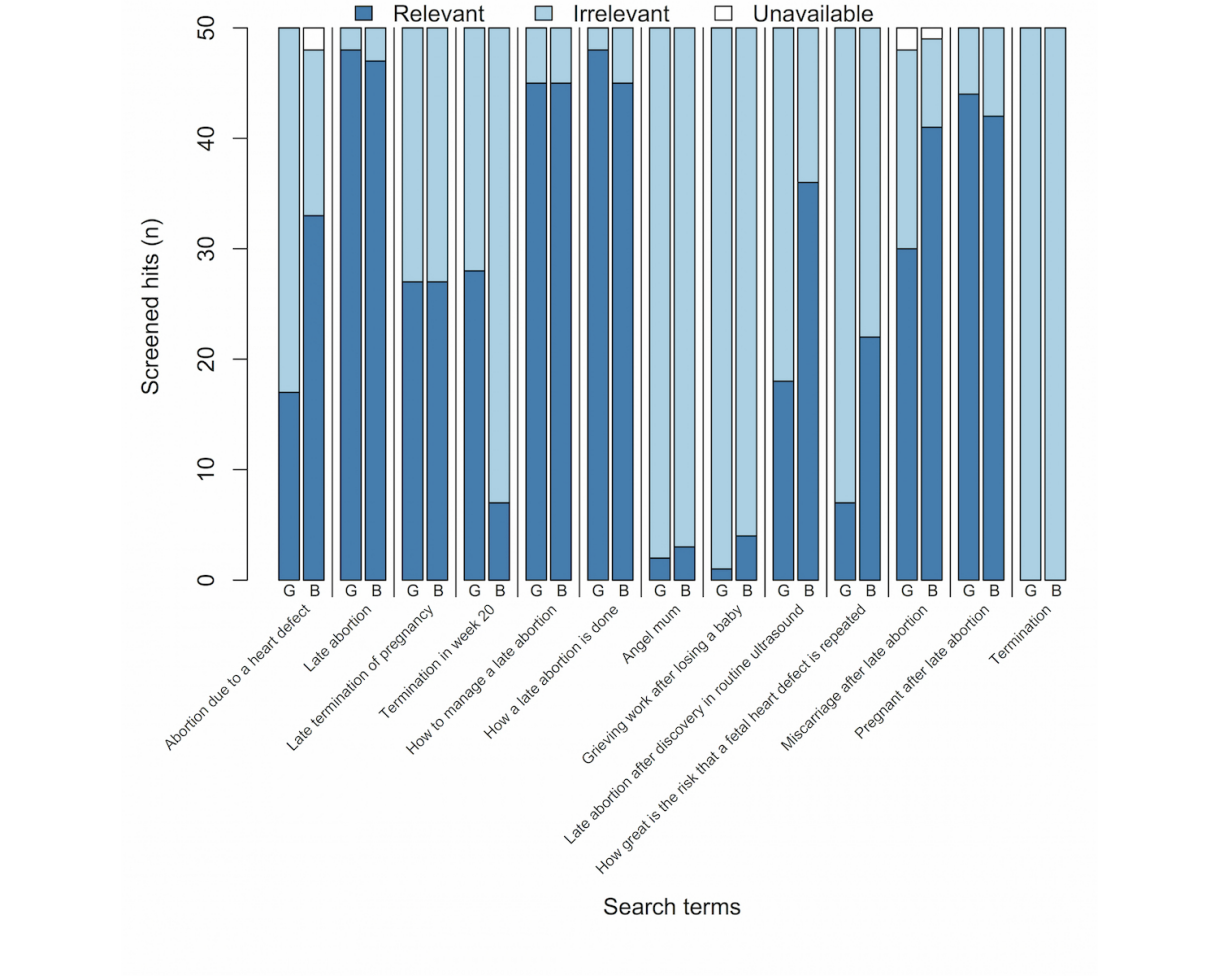
Number of relevant and irrelevant hits among the first 50 hits in Google (G) and Bing (B).

Figure 1 presents the sampling procedure. The first 50 hits of each search (N=1300 hits screened in total) were screened for inclusion by the first author. The 4 individuals who have experience with medically induced second-trimester abortions were not involved in the process of site selection. To be included, the websites needed to provide patient information about medically induced second-trimester abortions and be written in Swedish. In total, 48% (628/1300) of the hits were irrelevant, 5 hits were unavailable and 51% (667/1300) led to websites about medically induced second-trimester abortions. Figure 2 presents the number of relevant and irrelevant hits among the first 50 hits in Google and Bing.

Of the relevant hits, 64% (428/667) were excluded because they led to (1) virtual communities (136/667), (2) news websites (114/667), (3) blogs (96/667), (4) scientific reports (52/667), (5) websites intended for health professionals (19/667), (6) websites in other languages (8/667), or (7) video websites (3/667). This resulted in 18% (239/1300) of the total hits leading to patient information websites about medically induced second-trimester abortions. Of these, 82% (197/239) were duplicate hits. Consequently, 42 websites were included: 43% (18/42) of which were affiliated with the health care system, and 57% (24/42) with private organizations. More than half of the included websites were identified through searches in both Google and Bing (69%, 29/42). Seven of the included websites were only identified through searches in Google, and 6 were only identified through searches in Bing.

### Data Collection

The same 4 individuals who reported the search terms were also used as assessors of the included websites. These assessors with personal experience with medically induced second-trimester abortion were recruited from a consecutively recruited sample of women and partners participating in an interview study at 2 tertiary fetal medicine centers in central Sweden. From this original sample, 4 assessors were purposefully invited to participate in the assessments, with the aim of selecting a representation of males and females with different ages. Two assessors were females and 2 were male partners, all native Swedes between the ages of 23 and 42 years. Two of the assessors were a couple and the other 2 were not associated with one another. The assessors individually accessed and rated each of the included websites at their homes between November 2015 and January 2016, resulting in 42 assessments from each of the assessors (168 assessments in total). Because we aimed to investigate the perspectives of laypeople, the assessors did not receive any particular training for performing the assessments. They were instructed to access, read, and assess any sections of the included websites that they considered relevant and related to the topic of medically induced second-trimester abortions. The couple was instructed to assess the websites independently.

#### Website Quality

The DISCERN instrument [30] and the Ensuring Quality Information for Patients (EQIP) tool [31] were used to assess website quality; both are validated and reliable instruments for systematically assessing the quality of patient information [32-34]. The DISCERN instrument was chosen because it has been used extensively in previous studies, and the EQIP tool was chosen because it includes dimensions not covered by DISCERN, such as design and language [34]. The 2 instruments were translated into Swedish by a native Swedish speaker and back-translated by a native English speaker to check for consistency.

DISCERN contains 16 questions rated on a Likert scale from 1 (serious shortcomings) to 5 (minimal shortcomings), resulting in a total score between 16 and 80. The 3 sections assess reliability (8 questions), information about treatment (7 questions), and overall quality (1 question) [30]. EQIP contains 20 questions rated as “yes” (1 point, quality criterion fulfilled), “partly” (0.5 point, quality criterion partly fulfilled), and “no” (0 point, quality criterion not fulfilled). The final score is calculated as a percentage of the maximum achievable score, resulting in a total score between 0 and 100 [31].

#### Suitability

The assessors were asked if they would recommend the website to others (yes or no) and to rate the suitability of the website as a source of information for individuals awaiting a medically induced second-trimester abortion, on a Likert scale from 1 (very unsuitable) to 5 (very suitable).

#### Issues

One open-ended question was asked regarding perceived issues with the websites, in which the assessors were free to write as much or as little as they wanted in free text.

### Data Analysis

The data was analyzed using R version 3.2.2 (R Foundation for Statistical Computing). Intraclass correlation coefficients were calculated to determine interrater reliability. Independent *t* tests were used to compare the mean scores of websites from the health care system and private organizations; *P*<.05 was considered statistically significant.

The responses to the open-ended question were analyzed using manifest qualitative content analysis, a method that aims to systematically find patterns in written text [35]. Meaning units concerning issues with the websites were identified and defined as words, sentences, or paragraphs containing aspects related to each other through their content and context. These meaning units were organized into categories, that is, collections of meaning units that shared similar content.

### Ethical Considerations

The study was approved by the regional ethics committee in Uppsala, Sweden (Reference number 2014/504, approval date: 14/01/2015). Informed consent was collected before enrolment and the assessors received SEK 3000 (approximately US $350) for their work.

## Results

### Website Quality

The total mean score was 36 (SD 10) of a maximum achievable score of 80 for DISCERN and 40 (SD 14) of a maximum achievable score of 100 for EQIP. The interrater reliability for all 4 assessors ranged from 0.77 to 0.83 and was somewhat lower for the 2 assessors who were a couple (for the couple: total DISCERN=0.77, reliability=0.67, information about treatment=0.75, overall quality=0.83, total EQIP=0.55). Compared with websites from the health care system, websites from private organizations had significantly lower reliability and EQIP total scores (Table 2).

**Table 2:** Interrater reliability, assessment means, and standard deviations for the websites from the health care system (n=18) and private organizations (n=24).

Instrument or tool (maximum achievable score)		Interrater reliability	Health care system (n=18)	Private organizations (n=24)	Total (n=42)
			Mean (SD)	Mean (SD)	Mean (SD)
**DISCERN**					
	Reliability (40)	0.79	22.4^a^ (5.3)	19.6^a^ (5.8)	20.8 (5.7)
	Information about treatment (35)	0.77	11.9 (4.1)	14.0 (5.4)	13.1 (5.0)
	Overall quality (5)	0.83	2.2 (1.2)	2.1 (1.1)	2.2 (1.1)
	Total score (80)	0.80	36.5 (8.9)	35.7 (10.7)	36.0 (10.0)
**EQIP^b^**					
	Total score (100)	0.78	44.6^a^ (11.5)	36.6^a^ (15.3)	40.0 (14.3)

^a^*P*<.05.

^b^EQIP: Ensuring Quality Information for Patients.

**Figure 3:**
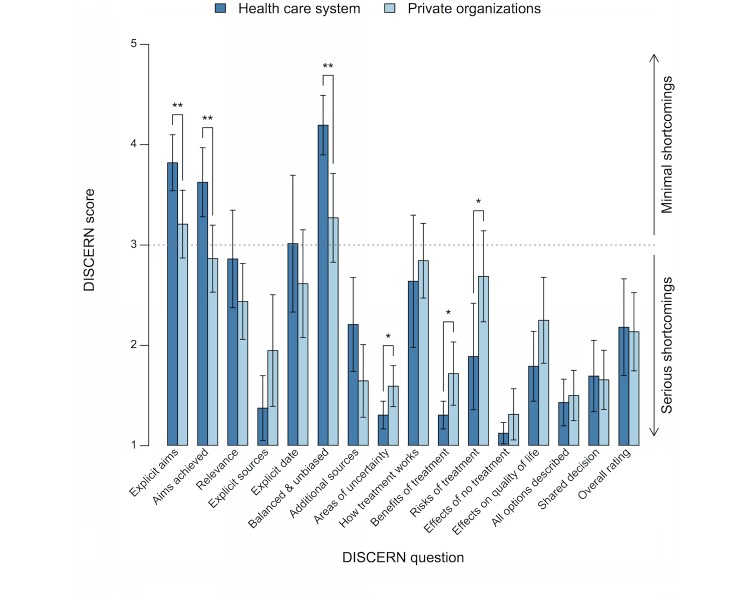
Mean scores and 95% CIs for each question in the DISCERN instrument. Comparisons are presented between websites from the health care system and private organizations (* *P*<.05, ** *P*<.01).

Mean scores and 95% CIs for each question in the DISCERN instrument are presented in Figure 3. Websites from both the health care system and private organizations had mean scores below 3.0 for 12 of the 16 questions, representing below moderate quality. Compared with websites from the health care system, websites from private organizations had significantly lower scores (*P*<.01) for the questions “explicit aims,” “aims achieved,” and “balanced and unbiased information.” Conversely, websites from private organizations had significantly higher scores (*P*<.05) for the questions “areas of uncertainty,” “benefits of treatment,” and “risks of treatment.”

**Figure 4:**
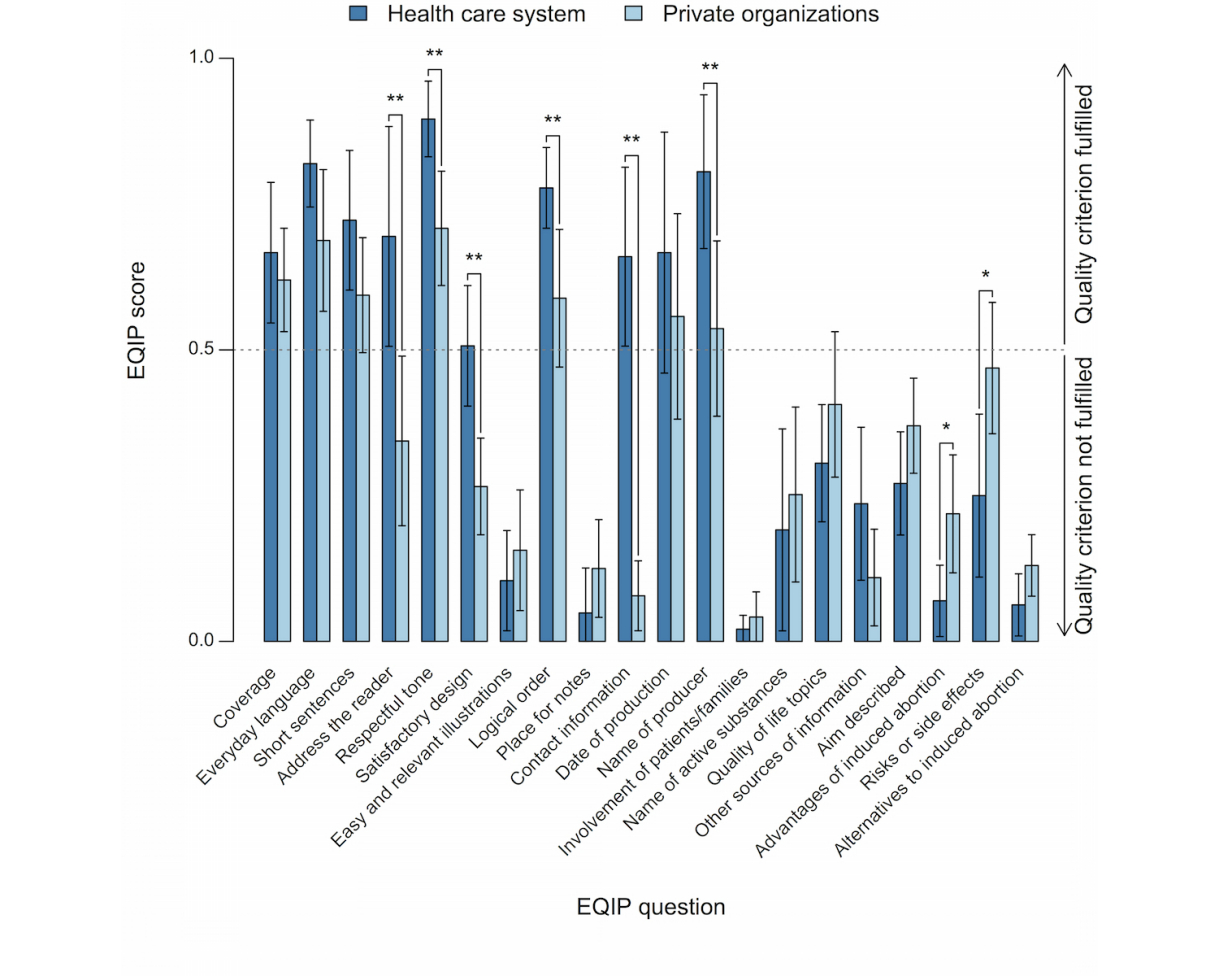
Mean scores and 95% CIs for each question in the Ensuring Quality Information for Patients (EQIP) tool. Comparisons are presented between websites from the health care system and private organizations (* *P*<.05, ** *P*<.01).

Mean scores and 95% CIs for each question in the EQIP tool are presented in Figure 4. Websites from both the health care system and private organizations had mean scores below 0.5 for 10 of the 20 questions, representing below moderate quality. Compared with websites from the health care system, websites from private organizations had significantly lower scores (*P*<.01) for the questions “address the reader,” “respectful tone,” “satisfactory design,” “logical order,” “contact information,” and “name of producer.” Conversely, websites from private organizations had significantly higher scores (*P*<.05) for the questions “advantages of induced abortion” and “risks or side effects of induced abortion.”

### Suitability

The mean score for suitability was 2.6 (SD 1.2) of a total score of 5.0, indicating below moderate suitability (corresponding to a score of 3.0). More than half of the websites (57%, 24/42) were rated as very unsuitable by at least one assessor. No significant differences in suitability scores were observed between websites from the health care system (mean 2.8/5.0, SD 1.1) and private organizations (mean 2.3/5.0, SD 1.2). Few websites were recommended by more than 1 assessor (31%, 13/42), and only 1 website was recommended by all 4 assessors (Table 3).

### Issues

Nine categories of issues were identified in total (Table 4). The most reported issues with the websites, reported by at least one assessor, were lack of information (76%, 32/42) and poor design (36%, 15/42). All websites from the health care system (n=18) had at least one assessor who reported lack of information, compared with 58% (14/24) from private organizations.

**Table 3:** Websites that were recommended by 1, 2, 3 or all assessors from the health care system (n=18) and private organizations (n=24).

Recommended by number of assessors (n=4)	Health care system (n=18)	Private organizations (n=24)	Total (n=42)
	n (%)	n (%)	n (%)
1 assessor	2 (11)	7 (29)	9 (21)
2 assessors	4 (22)	2 (8)	6 (14)
3 assessors	3 (17)	3 (13)	6 (14)
4 assessors	1 (6)	0 (0)	1 (2)

**Table 4:** Issues reported by at least one assessor, for websites from the health care system (n=18) and private organizations (n=24), identified in the open-ended question.

Reported issue		Health care system (n=18)	Private organizations (n=24)	Total (N=42)	Illustrative quote
		n (%)	n (%)	n (%)	
**Lack of information**		18 (100)	14 (58)	32 (76)	Hardly any information on the website
	The abortion procedure	10 (56)	5 (21)	15 (36)	Does not address the procedure
	Emotional difficulties	5 (28)	3 (13)	8 (19)	Scanty information on the emotional side of things
	Reasons for abortion	2 (11)	3 (13)	5 (12)	No info on reasons for late abortion
	Professional support	2 (11)	2 (8)	4 (10)	No info on what help is available
	The fetus	3 (17)	1 (4)	4 (10)	No mention at all of what happens to the fetus
	Medications	1 (6)	1 (4)	2 (5)	No info on how Mifepristone impacts the fetus
	Follow-up care	0 (0)	1 (4)	1 (2)	Nothing on aftercare
	No contact information for health care services	1 (6)	0 (0)	1 (2)	Does not say who to contact
Poor design		4 (22)	11 (46)	15 (36)	Disastrous interface
Disrespectful and belittling tone		4 (22)	5 (21)	9 (21)	It is like a sermon, and has a negative and arrogant tone
Poor language		1 (6)	8 (33)	9 (21)	Very difficult to understand what it says, as the words hardly form sentences
Biased against abortions		0 (0)	8 (33)	8 (19)	Biased website, clearly against abortion
Inaccurate information		0 (0)	5 (21)	5 (12)	Description of late abortion is completely wrong
Irrelevant information		2 (11)	3 (13)	5 (12)	No relevant information at all
Untrustworthy or unclear source of the information		0 (0)	3 (13)	3 (7)	No info on who produced the site

## Discussion

### Principal Findings

The included websites had poor quality and suitability, and the majority had issues with lack of information. Although the difference was small, websites from the health care system had higher reliability and overall EQIP quality scores. The Web contains an immense number of websites, resulting in an overwhelming amount of information and searching difficulties [14,36,37]. Moreover, it has been shown that consumers of health information use suboptimal search strategies [11] and are unsuccessful at finding satisfactory information on the Web [38]. The findings of this study confirm this, as the search terms reported by laypeople resulted in a high number of irrelevant hits and few hits leading to relevant patient information websites. Considering the small number of relevant patient information websites identified with these search terms, the findings indicate a need for health professionals to involve themselves by initiating discussions about search strategies and information on the Web. Health professionals should inform patients about the potential difficulties of identifying relevant high-quality patient information websites. One potential strategy to encourage patients to come in contact with high-quality sources could be to offer a list of appropriate search terms and recommended websites. However, most professionals lack the time needed to familiarize themselves with the Web and the quality of its various available sources [10,39], and articulate a number of difficulties when consulting Web-informed patients [39,40]. Consequently, individual professionals cannot be expected to have the knowledge, technical skills, and time to identify and stay updated about appropriate Web-based sources. Thus, it is possible that a need exists for overarching institutions responsible for stipulating updated lists of recommendations for high-quality patient information available on the Web. Most appropriately, such efforts should be made in collaboration with laypeople that have personal experience with pregnancy termination.

The results concerning low website quality echo previous studies in other health contexts [13,14]. It seems that, in addition to containing inaccurate and misleading information [22,24], websites about abortions are also of poor quality and lack information. It has previously been shown that health information seekers place greater trust in sources from official authorities [11]. In contrast with this, our results indicate that websites from both the health care system and private organizations have low reliability and poor-quality information about medically induced second-trimester abortions. A particular issue with the included websites was the lack of comprehensiveness. Most of the websites were reported to lack information about topics the assessors considered important, and both quality instruments indicated very poor quality concerning information about quality of life and risks of treatment. The results indicate that health professionals must make critical appraisals before referring patients to websites about medically induced second-trimester abortions, irrespective of website affiliation. Overall, there is a great need for systematic efforts to improve the quality of patient information websites about medically induced second-trimester abortions. Website developers must take steps to ensure sources that correspond with the preferences and needs of laypeople awaiting pregnancy termination. One such step could be to involve patients in the production of the information [27]. The included websites had low scores for the EQIP questions concerning involvement of patients, indicating a need for improvement.

The assessors recommended few of the included websites, the majority of the websites were considered unsuitable, and one-fifth were considered to have a disrespectful or belittling tone, including 4 from the health care system. Moreover, websites from private organizations had significantly lower scores for the EQIP question concerning whether the information was written in a respectful tone, suggesting that some of these websites may be particularly disrespectful. In accordance with our findings, previous research has highlighted that patients who read printed patient information criticize these materials by calling attention to the use of patronizing language, including a conveyed attitude that the doctor knows best [27]. Nonjudgmental and respectful attitudes are indicators of quality abortion care [20], and are highly desired by women who seek abortions [17,41]. Many women who terminate a pregnancy regard the decision as emotionally painful [16,17,42] and are at risk of significant psychological consequences [43]. Contact with unsuitable and disrespectful information could potentially increase these difficulties and aggravate psychological morbidity. Moreover, many of the included websites were criticized for use of poor language, which made it difficult to understand the information. Website and information developers should take note of these findings, and work toward the use of respectful and easy-to-read language about abortions.

### Strengths and Limitations

One strength of this study is that it used assessments by individuals with experience with medically induced second-trimester abortions. To our knowledge, no other study has included laypeople with personal experience to assess the quality of websites offering information about medically induced second-trimester abortions. Thus, this study investigated patient perspectives on information websites, leading to a more patient-focused approach. For example, the reported lack of information illustrates a mismatch between what information individuals want when awaiting a medically induced second-trimester abortion and the information produced by website developers. Moreover, the reported disrespectful or belittling tone highlights the strength of using laypeople as assessors. These findings call attention to the importance of consumer-focused perspectives in studies investigating website quality.

Only Swedish websites were included, which could implicate limited generalizability to other countries with different contexts. However, Swedish websites are probably similar to those of many other countries. To identify websites accessed by laypeople, search terms from individuals with personal experience were used. For each search term, the first 50 hits were screened for inclusion, resulting in 1300 screened hits in total. Previous research has shown that individuals rarely go beyond the first 10 hits [11]. Moreover, many of the screened hits that led to relevant patient information websites were duplicate hits, indicating that we achieved saturation. Google and Bing are the 2 most used search engines in Sweden, Google being the most used, with over 90% of usage [44]. The majority of the included websites were identified through searches in both Google and Bing. Considering these aspects, we argue that the patient information websites identified represent those that women and their partners come in contact with when searching the Web for patient information about medically induced second-trimester abortions.

Two validated instruments [32-34] developed by patient information experts and laypeople were used to assess the quality of the included websites. The DISCERN instrument has been used extensively in previous studies to assess the quality of information on the Web about many different health issues, such as congenital heart defects [14] and caesarean section [45]. The EQIP tool was chosen since it includes dimensions not covered by DISCERN, such as design and language [34], and has previously been used to assess, for example, the quality of websites offering information on breast augmentation [46]. Thus, the 2 instruments complemented one another and showed similar results concerning poor website quality. The instruments were originally developed to systematically judge the quality of patient information [32,33] and the interrater reliabilities among the assessors were approximately 0.8 across the different subscales, indicating substantial agreement [47]. A separate analysis of the ratings of the 2 assessors who were a couple did not reveal higher interrater reliability compared with the overall interrater reliability. Consequently, we argue that the couple rated the websites individually and that the inclusion of a couple as assessors did not introduce bias into the study.

### Suggestions for Future Research

This was a descriptive study with the overarching aim to investigate the quality of, suitability of, and issues with existing patient information websites about medically induced second-trimester abortions. The findings highlight a number of different problems that exist with these sources, calling attention to further studies needed within this field of research. Steps need to be taken to raise the overall quality of patient information websites about medically induced second-trimester abortions.

First, the confronted searching difficulties highlight the need to investigate which counseling strategies are appropriate to guide consumers to the highest quality Web-based sources. Second, more research is needed that investigates the potential effects that publicly available patient information websites have on the psychological health of individuals awaiting medically induced second-trimester abortions. Finally, the findings call attention to the mismatch between the preferences of laypeople and existing websites, indicating a need for research that investigates how websites should be developed to meet the requirements of the intended consumers. For example, the next step could be to develop guidelines for website development for this specific patient population. We encourage researchers to initiate intervention studies with the aim of raising the quality standard of the available sources on the Web about medically induced second-trimester abortions.

### Conclusions

The high number of irrelevant hits and poor quality of patient information websites are issues that must be considered when consulting patients awaiting medically induced second-trimester abortions. Poor quality of the information and low reliability were found in websites affiliated with the health care system as well as private organizations, indicating problems irrespective of website affiliation. Although the difference was small, websites from the health care system had higher reliability and overall quality.

When consulting women and partners who are awaiting medically induced second-trimester abortions, health professionals should initiate discussions concerning web-based patient information sources, and inform them about the issues and quality deficits associated with these websites. In clinical encounters, professionals should offer recommendations for appropriate search terms, search strategies, and patient information websites. There is a need for overarching systematic efforts to stipulate and continuously update lists with such recommendations.

The results indicate that website developers need to take steps to enhance the quality of websites about medically induced second-trimester abortions and ensure that websites meet the preferences and needs of the intended consumers. Developers should make sure that websites contain comprehensive, accurate, and easy-to-read information. The information must be written respectfully and without bias against induced abortions, and include details about how the information was produced. More research is needed to investigate how to help patients come in contact with the most appropriate web-based supplemental patient information about medically induced second-trimester abortions.

## Abbreviations

EQIP: Ensuring Quality Information for Patients
